# Author Correction: Ultra-thin enzymatic liquid membrane for CO_2_ separation and capture

**DOI:** 10.1038/s41467-018-04642-6

**Published:** 2018-06-01

**Authors:** Yaqin Fu, Ying-Bing Jiang, Darren Dunphy, Haifeng Xiong, Eric Coker, Stan Chou, Hongxia Zhang, Juan M. Vanegas, Jonas G. Croissant, Joseph L. Cecchi, Susan B. Rempe, C. Jeffrey Brinker

**Affiliations:** 1 Department of Chemical and Biological Engineering, University of New Mexico, Albuquerque, NM 87131 USA; 2 Center for Micro-Engineered Materials, University of New Mexico, Albuquerque, NM 87131 USA; 3 Department of Earth and Planetary Sciences, University of New Mexico, Albuquerque, NM 87131 USA; 4 Sandia National Laboratories, Albuquerque, NM 87185 USA; 5 Angstrom Thin Film Technologies LLC, Albuquerque, NM 87113 USA; 6 Department of Physics, University of Vermont, Burlington, VT 05405 USA

Correction to: *Nature Communications* 10.1038/s41467-018-03285-x, published online 07 Mar 2018

The original version of this Article contained an error in the spelling of the author Stanley S. Chou, which was incorrectly given as Stan Chou. This has now been corrected in both the PDF and HTML versions of the Article.

