# Supplementary material for: Ultra-thin enzymatic liquid membrane for CO2 separation and capture
Source: Nat Commun. 2018 Mar 7;9:990. doi: 10.1038/s41467-018-03285-x (PMC5841395; doi:10.1038/s41467-018-03285-x)
Supplement: Supplementary file 1 — Supplementary Information [file 41467_2018_3285_MOESM1_ESM.pdf]

Supplementary Information

Ultra-thin nano-stabilized enzymatic liquid membrane for CO_2 separation and capture

Yaqin Fu[1,2], Ying-Bing Jiang[*,1,2,3], Darren Dunphy[1,2], Haifeng Xiong[1,2], Eric Coker[4], Stan Chou[4], Hongxia Zhang[5], Juan M. Vanegas[4], Jonas G. Croissant[1,2], Joseph L. Cecchi[1], Susan B Rempe[4] & C. Jeffrey Brinker[*,1,2,4]

TABLE OF CONTENT

Supplementary Figure 1 | Enzymatic active site structure during the catalytic cycle……… **p. 2**

Supplementary Figure 2 | Microscopy images of the Anodisc support…….……………..… **p. 3**

Supplementary Figure 3 | CO_2/N_2 separation set-up…………………………………....… **p. 4**

Supplementary Figure 4 | CO_2/N_2 gas chromatography analysis…………………………… **p. 5**

Supplementary Figure 5 | CO_2/H_2 separation set-up…………………………………....… **p. 6**

Supplementary Figure 6 | Selectivity of the CO_2/H_2 separation as a function of time……… **p. 7**

Supplementary Figure 7 | Stability of the enzymatic liquid membrane overtime………...... **p. 8**

Supplementary Discussion………………………………………………….…………………… **p. 9**

Supplementary Discussion…………………………………………………………...……… **p. 10**

Supplementary Discussion…………………………………………………………...… **p. 11**

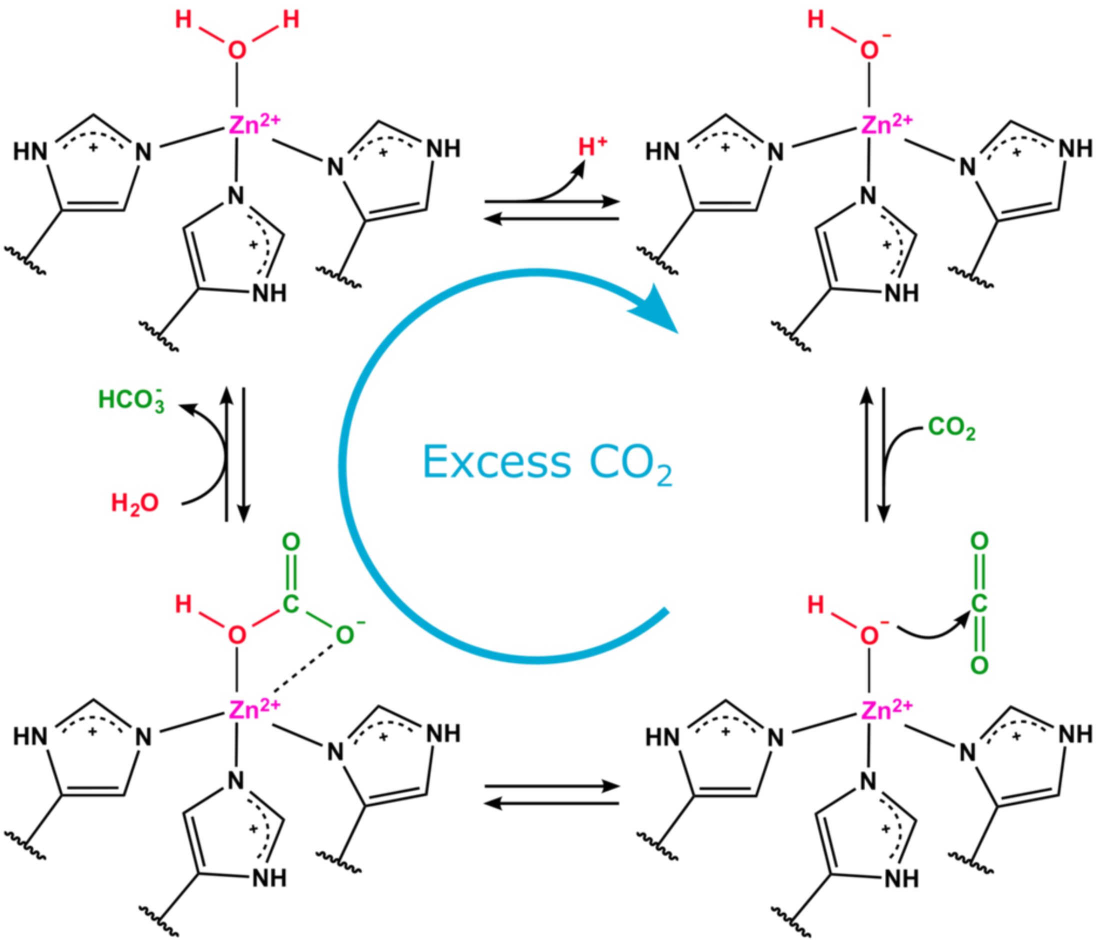

Supplementary Figure 1 | Enzymatic active structure site during the catalytic cycle. Key reactions catalyzed by carbonic anhydrase enzyme, viewed from the active site. A zinc ion (Zn^{2+}) coordinated with three histidine residues greatly enhances the acidity of the zinc-bound water molecule. A chain of protonatable residues shuttles the water proton out of the active site to avoid back-reaction. The active site orients CO_2 near the deprotonated water (OH^-). As a result, the enzyme dramatically accelerates CO_2 hydration to bicarbonate ion (HCO_3^-), even when the solution is neutral.

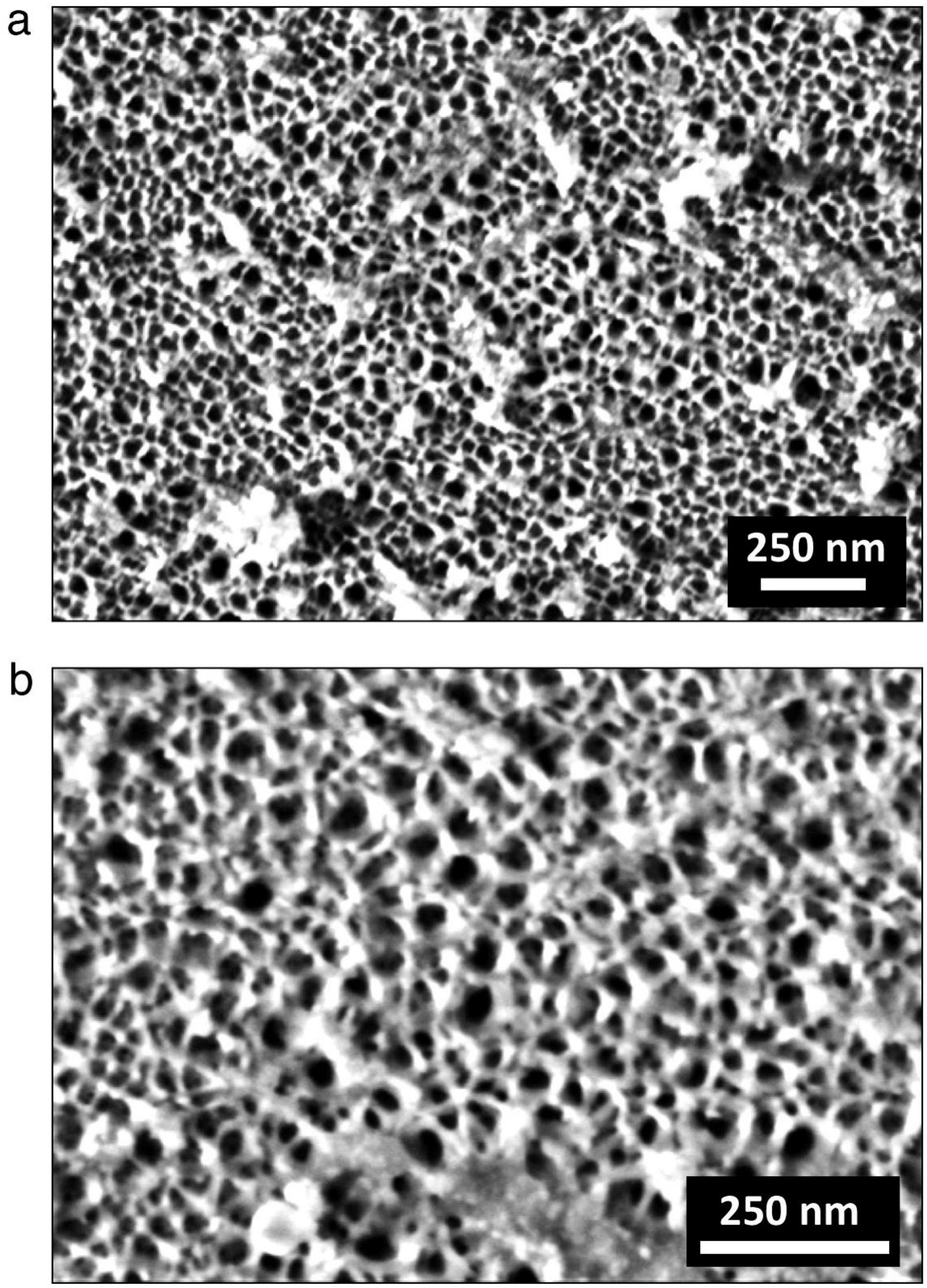

Supplementary Figure 2 | Microscopy images of the Anodisc support. (**a-b**) Plan-view SEM images of the Anodisc at two magnification scales showing the size distribution of the Anodisc mesopores, which constitute ca 70% of the Anodisc surface.

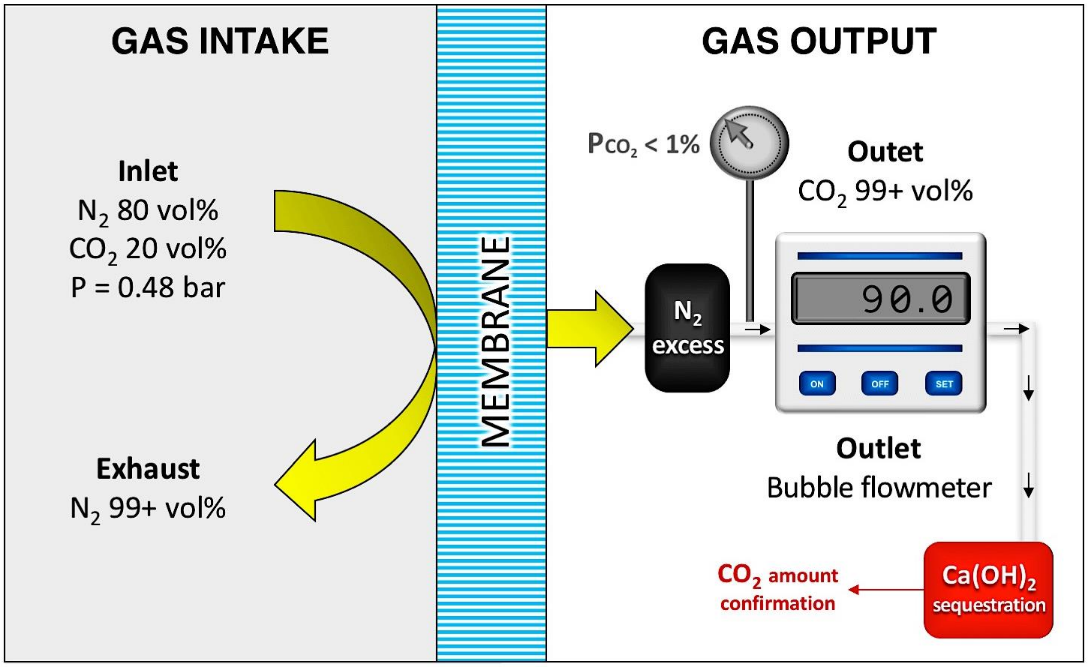

Supplementary Figure 3 | CO_2/N_2 separation set-up. On the gas intake side, the feed gas composition was 20 vol% CO_2 in N_2 maintained at an ambient pressure in order to simulate the major composition of flue gas from a coal-fired plant. On the gas output side, a bubble flow rate meter was used to measure the flow rate, and the CO_2 pressure was maintained at ambient atmospheric pressure. An excess amount of pure N_2 was pre-stored in order to keep the CO_2 partial pressure below 1% during the bubble flowmeter measurement. A $Ca(OH)_2$ solution was used to collect and determine the permeated CO_2 content and maintain a constant chemical potential driving force for CO_2 separation.

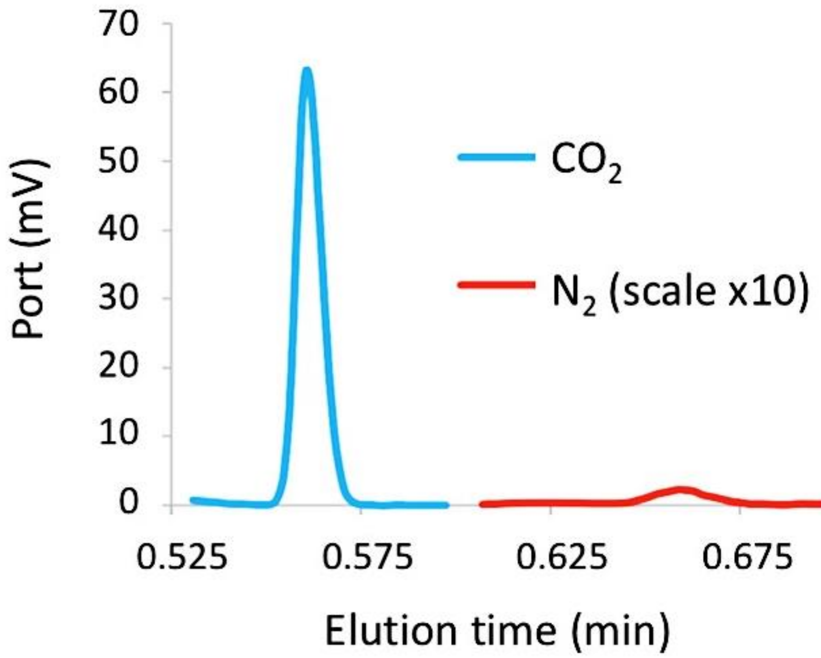

Supplementary Figure 4 | CO_2/N_2 gas chromatography analysis. Gas chromatograph showing composition of the output gas through the enzymatic liquid membrane, plotted as signal strength as a function of time, when the input feed is a 1:1 mixture of CO_2 and N_2 gas. Here, the CO_2 to N_2 selectivity is above 750.

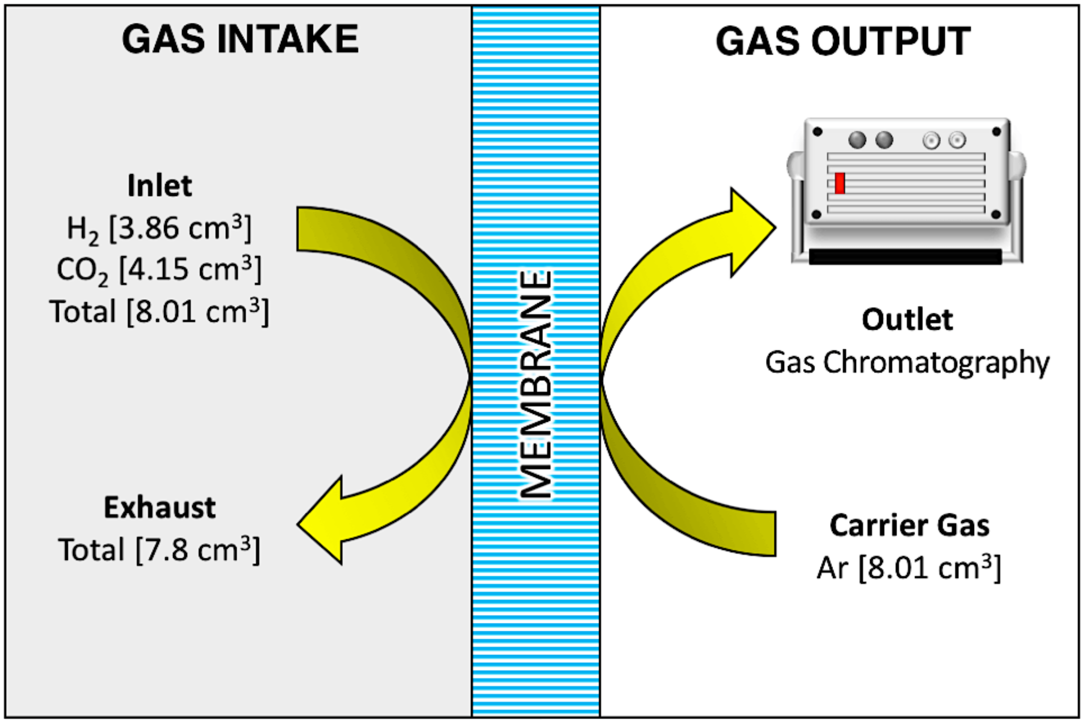

Supplementary Figure 5 | CO_2/H_2 separation set-up. A cross flow configuration was used for H_2 permeation measurements. Feed gas composition was fixed at 43% H_2 and 57% CO_2. The quantity of gas permeating across the membrane was calculated by the difference in gas flow at the inlet versus the exhaust, with a typical cross-flow rate of 0.21 cm^3. Gas permeated across the membrane was then carried by an Ar gas (8.01 cm^3) into a calibrated Inficon 3000 Micro GC gas analyzer for quantitative measurement discrimination.

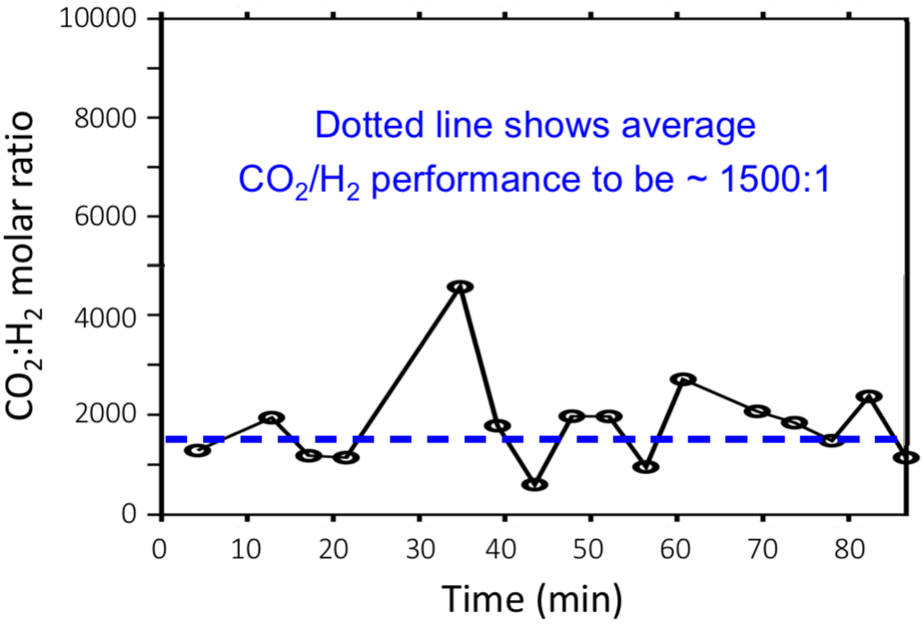

Supplementary Figure 6 | **Selectivity of the CO_2/H_2 separation as a function of time.** Each data point was acquired via a gas chromatography run.

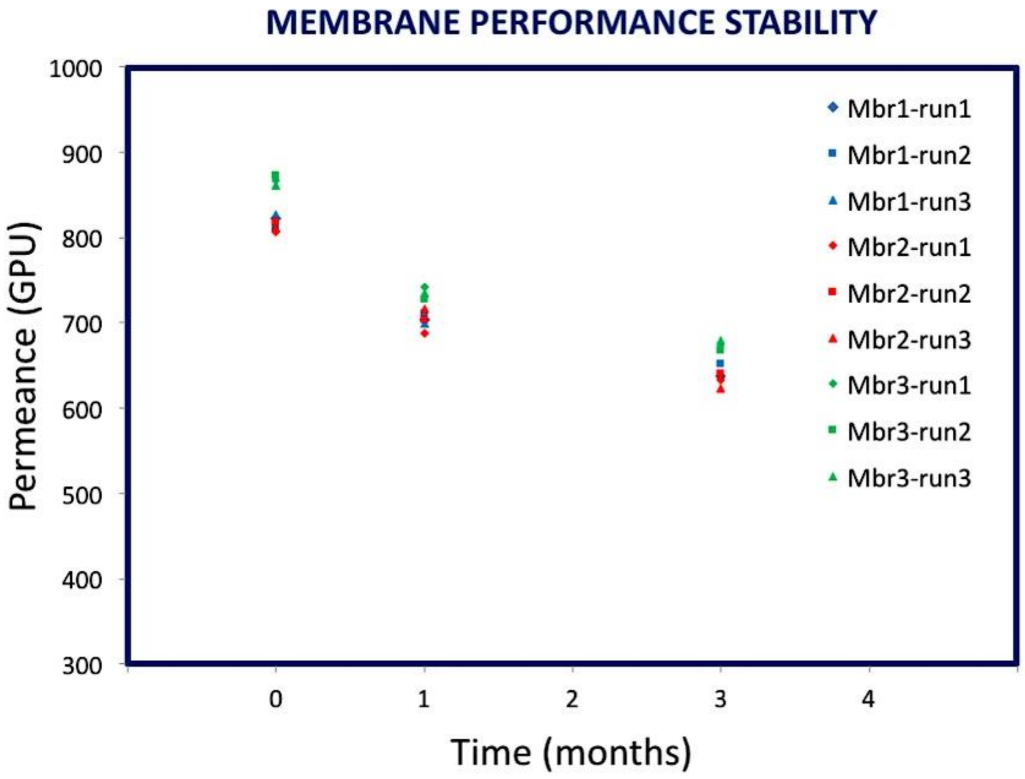

Supplementary Figure 7 | Stability of the enzymatic liquid membrane overtime maintained at >90%RH. The CO_2 permeance of the enzymatic liquid membranes (N=3) shows a moderate and limiting loss of permeance over 3 months, but the permeance exceeds that of most polymeric membranes and the CO_2/N_2 selectivity remains above 500.

SUPPLEMENTARY DISCUSSION

❖ DETERMINATION OF THE RELATIVE HUMIDITY NECESSARY FOR THE STABILITY OF MEMBRANES WITH VARIOUS PORE SIZES

$$\mathrm{Ln}\frac{P_v}{P_{sat}} = -\frac{2H\,\gamma\,V_m}{RT}$$

$$\mathrm{Ln}(RH) = -\frac{2\,\gamma\,V_m}{r\,RT}$$

$$= -\frac{2 \times 0.072\,(\mathrm{N\,m^{-1}}) \times 18\,(\mathrm{cm^3\,mol^{-1}})}{r \times 8.32\,(\mathrm{J\,mol^{-1}\,K^{-1}}) \times 298\,(\mathrm{K})}$$

r = 3 nm (6 nm pores); RH = 65.2 %

r = 4 nm (8 nm pores); RH = 73.9 %

P_v : equilibrium vapor pressure

P_{sat} : saturation vapor pressure

RH : relative humidity

H : average meniscus curvature

γ : liquid-air surface tension

V_m : liquid molar volume

R : ideal gas constant

T : temperature

r : radius of the membrane

❖ CONCLUSION

Will the membrane dry out under the operation conditions? No, 6 to 8 nm-wide highly hydrophilic pores capture and hold water at about 70% humidity. A typical flue gas comprises 6.2 wt% H_2O if it is from a coal-fired plant, and 14.6 wt% H_2O if it is from a gas-fired plant. Both are much higher than the saturated water vapor concentration at 40 °C (~50g H_2O /kg air or 0.5 wt% H_2O).

SUPPLEMENTARY DISCUSSION

❖ NANOPORE CAPILLARY PRESSURE EQUATION FOR WATER CONDENSED IN A HYDROPHILIC PORE WITH LIQUID CONTACT ANGLE 0

$$P = \frac{2\gamma \, . \cos\theta}{d} = \frac{0.144 \text{ (Pa)}}{d \text{ (m)}}$$

$$= \frac{1.42 \text{ (atm)}}{d \text{ (}\mu\text{m)}}$$

$$= \frac{2.8 \text{ (atm)}}{d \text{ (}\mu\text{m)}}$$

P : pressure on the membrane

d : diameter of the membrane

γ : liquid-air surface tension

θ : liquid contact angle

d = 1 μm (1 μm pores); P = 2.8 atm

d = 10 nm (10 nm pores); P = 28 atm

d = 8 nm (8 nm pores); P = 35 atm

❖ CONCLUSION

Will the membrane be stable under higher pressures? Yes, because of the capillary pressure, the enzymatic liquid membrane can withstand tens of atmospheres of pressure and should not be displaced from the membrane under operation.

SUPPLEMENTARY DISCUSSION

❖ DETERMINATION OF THE AREAL DENSITY OF 8 nm SILICA MESOPORES EXPOSED ON THE ANODISC SUPPORT SURFACE

Step 1: Determine the areal density of mesopores in individual Anodisc pores by image analysis.

Procedure: direct TEM imaging of mesopore arrays within Anodisc pores and determination of the numbers of mesopores per cm^2.

Result: 5.60×10^{11} nanopores per cm^2.

Step 2: Determine of areal fraction of pores in Anodisc membrane support.

Procedure: direct SEM imaging of Anodisc surface (Supplementary Fig. S3) and determination of fraction porosity by random line cut analysis.

Result: 70%.

Step 3: Assuming that all Anodisc pores are completely filled with silica mesopores with a density of 5.60×10^{11} nanopores per cm^2, we calculated the areal density of silica mesopores exposed on the Anodisc surface. Result: $0.7 \times 5.60 \times 10^{11} = 3.92 \times 10^{11}$ nanopores per cm^2.

❖ CONCLUSION

The overall areal density of the alumina Anodisc support-nanoporous silica is of 3.92×10^{11} nanopores per cm^2.
